# Conducting botanical research with limited resources: Low‐cost methods in the plant sciences

**DOI:** 10.1002/aps3.11341

**Published:** 2020-04-20

**Authors:** Gillian H. Dean, Alice N. Muriithi, Theresa M. Culley

**Affiliations:** ^1^ Department of Botany University of British Columbia 6270 University Boulevard Vancouver V6T 1Z4 Canada; ^2^ School of Agricultural and Food Sciences Jaramogi Oginga Odinga University of Science and Technology Box 210-40601 Bondo Kenya; ^3^ Department of Biological Sciences University of Cincinnati 614 Rieveschl Hall Cincinnati Ohio 45221‐0006 USA

Substantial resource barriers exist for many researchers that hamper their efforts to generate high‐quality data that can be successfully published. Globally, considerable scientific research is performed in under‐resourced settings, including in countries with less well‐developed research infrastructure, or by investigators in smaller colleges without access to expensive equipment or substantial funding. In addition, research is also being performed by novices, such as graduate students at the start of their careers, and researchers exploring new areas using unfamiliar techniques who are not able to invest in expensive equipment and start‐up costs. Finally, efforts to share expensive instruments between institutions in developing countries can be hampered by bureaucracy that causes significant delays to research, and even if equipment is purchased as part of a funded project, salary to employ a qualified technician and funds for upkeep after the project ends are often not included. This can result in specialized equipment not being used at all, or being decommissioned.

One way to build capacity and enable scientists from a diverse array of backgrounds to contribute to the scientific community is to develop and share robust, reliable, low‐cost protocols that can be used as alternatives to the more expensive methods and approaches commonly used in the plant sciences. The aim of this special issue is to provide a collection of methods that fit these criteria. While we were particularly interested in innovative protocols that can be used by scientists in less developed countries, we expect these methods will be valuable to a wide range of researchers as they can be employed in a variety of settings without complex and specialized equipment or reagents. Our scope for this special issue was broad, and is reflected in the diversity of methods covered here.

In some fields, hi‐tech methods that offer advantages in efficiency and data generation have replaced traditional botanical methods, even though these methods are more expensive and require greater investment in equipment and reagents. In this special issue, Windham et al. (2020) provide a detailed protocol for meiotic chromosome counts, a technique that has been underutilized in recent years but requires minimal lab equipment, and is simple and very effective once mastered. Similarly, correlations between spore size and genome size in ferns can be used to develop estimates for relative genome size, with enough resolution to infer ploidy (Barrington et al., 2020), allowing evolutionary history to be explored with minimal equipment and training.

Phenological observations to examine the timing of seasonal biological events using twig cutting and common garden experiments are relatively straightforward, low‐cost methods that can be used to dissect factors driving changes in phenology and probe the underlying mechanisms (McDonough MacKenzie et al., 2020). These phenological methods are of particular interest in the current period of climate change, and also offer opportunities to expand data collection by incorporating citizen science into research programs.

Other cost‐effective methods that are presented here include a simple density‐based protocol for separating seeds with different oil contents using salt solutions (Dean et al., 2020), development of mini‐rhizotrons built from CD cases for examining root phenotypes and root‐associated symbioses (Cassidy et al., 2020), a method to measure cyanide release from cyanogenic plants (Smiley and Morrison, 2020), a technique to extract xylem sap from drought‐stressed or bacterial wilt–infected tomato plants (Longchar et al., 2020), and a protocol to build a pump for sampling volatile organic compounds (Saryan and Gowda, 2020).

The use of genome‐scale sequence data for phylogenetic analysis (phylogenomics) and population genomics is one area that is developing rapidly due to advances in high‐throughput sequencing (HTS) technologies (McKain et al., 2018). Although the cost of these techniques has decreased greatly, they are still relatively expensive and therefore inaccessible for many researchers. However, two papers in this special issue highlight cost savings that can be incorporated into target capture (Hale et al., 2020) and double‐digest restriction site–associated DNA sequencing (ddRADseq; Jordon‐Thaden et al., 2020) workflows. In both papers, the authors assess the suitability of herbarium specimens for HTS, which also allows significant cost savings in terms of fieldwork.

The emerging field of plant microbiome research is another area where HTS is being used extensively. Johnston‐Monje and Lopez Mejilla (2020) demonstrate the use of cost‐effective molecular fingerprinting techniques for studying plant‐associated microbial populations, and show that these methods are still relevant for identifying dominant microbes in a population, observing changes in plant microbiome community diversity, and screening samples before embarking on more costly HTS.

We expect that this special issue will inspire researchers in plant biology to innovate and develop low‐cost methods that can be shared among scientists, and ultimately lead to greater equality in the scientific community.
